# Pemphigus and pemphigoid are associated with Alzheimer’s disease in older adults: evidence from the US Nationwide inpatient sample 2016–2018

**DOI:** 10.1186/s12877-023-04580-z

**Published:** 2023-12-19

**Authors:** Zhen Xie, Yang Gao, Lidi Tian, Yang Jiang, Hao Zhang, Yang Su

**Affiliations:** 1 Department of Dermatology, Sichuan Provincial People’s Hospital, University of Electronic Science and Technology of China, Chengdu, China; 2 General Practice Center, Sichuan Provincial People’s Hospital, University of Electronic Science and Technology of China, Chengdu, China; 3 Department of Dermatology, Ya’an People’s Hospital, 358 Chenghou Road, Yucheng District, Ya’an, China; 4 Department of Dermatology, Baoan Central Hospital of Shenzhen, 518001, No.6 Xinyuan Street, Xixiang Road, Shenzhen, 518001 China; 5 Department of Laboratory Medicine and Sichuan Provincial Key Laboratory for Human Disease Gene Study, Sichuan Provincial People’s Hospital, University of Electronic Science and Technology of China, Chengdu, China

**Keywords:** Alzheimer’s disease (AD), Pemphigus, Pemphigoid, Nationwide inpatient sample (NIS)

## Abstract

**Background:**

Pemphigus and pemphigoid are rare autoimmune skin disorders caused by autoantibodies against structural proteins and characterized by blistering of the skin and/or mucous membranes. Associations have been noted between skin diseases and Alzheimer’s dementia (AD). Dementia is a neurological disorder of progressive cognitive impairment with increasing incidence among older adults. This study aimed to assess the potential associations between pemphigus, pemphigoid and AD in a large, nationally representative US cohort.

**Methods:**

All data of hospitalized patients aged 60 years or older were extracted from the US Nationwide Inpatient Sample (NIS) database 2016–2018. Patients with a history of head trauma, diagnosis of vascular dementia, history of cerebrovascular disease, or malformation of cerebral vessels were excluded. The study population was divided into those with and without pemphigus (cohort 1) and with and without pemphigoid (cohort 2).

**Results:**

Pemphigus was independently associated with a 69% increased risk of AD. Adults ≥80 years old with pemphigus were 72% more likely to develop AD than adults without pemphigus. Women with pemphigus were 78% more likely to develop AD than women without pemphigus. On the other hand, pemphigoid was independently associated with a 39% increased risk for AD and subjects ≥80 years with pemphigoid were 40% more likely to have AD than those without pemphigoid. Females with pemphigoid were 63% more likely to have AD than those without pemphigoid. Moreover, Hispanic older adults with pemphigus were 3-times more likely to have AD than those without pemphigoid.

**Conclusions:**

Pemphigus and pemphigoid were both independently associated with AD in older adults, especially among females and octogenarians. Further studies addressing the etiology and mechanisms underlying these associations are highly warranted.

## Background

Alzheimer’s disease (AD), the most common type of dementia, is defined as a slowly progressive neurodegenerative disorder characterized by the accumulation of amyloid-β peptide (Aβ), which leads to formation of neuritic plaques (also known as amyloid-β plaques) and neurofibrillary tangles in the medial temporal lobe and neocortical structures [1]. Since the first case was reported by Alois Alzheimer in 1907, and despite ongoing research and great advances in our understanding of AD pathogenesis, no disease-improving treatments are yet available [2, 3]. The number of people living with dementia worldwide is estimated to exceed 45 million, which makes AD the most common form of dementia, accounting for 60–80% of all dementia cases [4]. Due to population aging and increased life expectancy, and also because multimodal treatments for AD remain unsuccessful, AD cases are expected to double by 2050 and the incidence rate to increase by 40% [5, 6].

AD is a complex, multifactorial disease determined by the interaction of genetic susceptibility and environmental factors encountered throughout life [7, 8]. To date, the identified modifiable risk factors for AD have been mainly associated with lifestyle factors or cardiovascular risk factors, including the presence of diabetes, hypertension, and obesity. It has been estimated that as many as one third of AD cases may be attributable to modifiable factors and therefore preventable [9].

Pemphigus vulgaris and bullous pemphigoid are relatively rare autoimmune skin disorders, while simultaneously being the most common autoimmune bullous diseases with reported increasing incidence [10, 11]. Autoimmune bullous diseases are a heterogeneous group of blistering skin diseases caused by autoantibodies formed against target antigens in the skin and mucous membranes [12]. The autoantibodies disrupt keratinocyte adhesion and cell integrity, resulting in the eruption of blisters. Pemphigus, or intraepidermal autoimmune bullous disease, is characterized by the formation of autoantibodies against desmoglein (Dsg), an intercellular adhesion molecule of keratinocytes [13, 14]. Pemphigoid or subepidermal autoimmune bullous disease, develops autoantibodies against components of the basement membrane zone, which impairs dermal-epidermal adhesion. While both disorders may occur in children and young adults, pemphigoid is more common among older adults [15].

Accumulating evidence has suggested that pemphigus and pemphigoid may be systemic immune-mediated disorders rather than isolated cutaneous immune disorders. Previous studies have shown an association between pemphigus, pemphigoid, and systemic diseases such as hypertension, obesity, dyslipidemia, metabolic syndrome, and insulin resistance, as well as autoimmune diseases such as lupus erythematosus [16]. Patients with bullous pemphigoid are more likely to develop neurological disorders, including cerebrovascular disease, Parkinson’s disease, and multiple sclerosis [17]. Previous studies have reported that several neurological disorders with central nervous system inflammation or degeneration, including epilepsy, Parkinson’s disease, and multiple sclerosis, are associated with pemphigoid [18, 19]. Recently, significantly high levels of pemphigoid antibodies were detected in stroke patients [20]. The role of neuropsychiatric symptoms in progression to dementia is not well understood [21]. Although neuropsychiatric symptoms are known to increase mortality risk in patients with AD, it is not known precisely if the presence of such symptoms, mild cognitive impairment or any specific syndrome increases this risk [22]. Meanwhile, it also remains unclear whether pemphigus and pemphigoid, which are both shown to have comorbid neurocognitive symptoms, are associated with AD. In this study, we aimed to evaluate the potential associations between pemphigus, pemphigoid and AD in a large, nationally representative inpatient population.

## Methods

### Data source

This population-based, cross-sectional study extracted all data from the US Nationwide Inpatient Sample (NIS) database, which is the largest all-payer, continuous inpatient care database in the United States, including about 8 million hospital stays each year. The database is administered by the Healthcare Cost and Utilization Project (HCUP) of the US National Institutes of Health (NIH) (https://www.hcup-us.ahrq.gov/db/nation/nis/NIS_Introduction_2020.jsp). Patient data include primary and secondary diagnoses, primary and secondary procedures, admission and discharge status, patient demographics, expected payment source, duration of hospital stay, and hospital characteristics (i.e., bed size/location/teaching status/hospital region). All admitted patients are initially considered for inclusion. The continuous, annually updated NIS database derives patient data from about 1050 hospitals from 44 States in the US, representing a 20% stratified sample of US community hospitals as defined by the American Hospital Association.

### Ethics statement

All data were obtained through request to the Online Healthcare Cost and Utilization Project (HCUP) Central Distributor, which administers the database. This study conforms to the NIS data-use agreement with HCUP (certification: #HCUP-5XW08I92F). Because this study analyzed secondary data from the NIS database, patients and the public were not involved directly. The study protocol was submitted to the Institutional Review Board (IRB) of Sichuan Provincial People’s Hospital, which exempted the study from IRB approval. Since all data in the NIS database are de-identified, the requirement for informed consent of patients was also waived.

### Study population selection

Data of patients aged 60 years or older who were hospitalized between 2016 and 2018 were identified in the NIS database. Subjects with history of head trauma, diagnosis of vascular dementia, history of cerebrovascular disease, or malformation of cerebral vessels, identified through corresponding ICD-10 codes in their medical record, were excluded. The study population was then divided into subjects with and without pemphigus (cohort 1), and subjects with and without pemphigoid (cohort 2) for subsequent data analysis. Pemphigus and pemphigoid were identified using the International Classification of Diseases, Tenth Revision, Clinical Modification (ICD-10-CM) codes: L10 (pemphigus); L12.0, and L12.8 (pemphigoid). The codes for identifying pemphigus and pemphigoid have been previously validated and regarded to have excellent specificity and positive predictive value in the hospital setting of the United States [23]. Furthermore, this approach has also been employed in several recently published studies [24, 25]. Subjects diagnosed with AD were identified using the ICD-10-CM codes G30.

### Covariates

Patients’ demographic characteristics included age, sex, race/ethnicity, household income, primary payer (insurance status). Patients’ clinical characteristics included chronic diseases/comorbidities (ischemic heart disease, valvular heart disease, diabetes mellitus [15], hypertension, dyslipidemia, obesity, chronic pulmonary disease, severe liver disease, moderate to severe kidney disease, coagulopathy, peripheral vascular disease, atrial fibrillation, psychosis, depression, systemic autoimmune disease, and other chronic inflammatory skin diseases), active tobacco use, and excessive alcohol use. Hospital-related characteristics (bed size, location/teaching status, and hospital region) were also extracted from the database as part of the comprehensive data available for all included participants.

### Statistical analysis

Since the NIS database covers 20% of samples of the USA annual inpatient admissions, weighted samples (before 2011 using TRENDWT & after 2012 using DISCWT), stratum (NIS_STRATUM), cluster (HOSPID) were used to produce national estimates for all analyses. The SURVEY procedure was applied to perform the analysis for the sample survey data. Descriptive statistics are presented as number (n) and weighted percentage (%) or mean and standard error (SE). Categorical data were analyzed using PROC SURVEYFREQ statement and continuous data were analyzed using PROC SURVEYREG statement. The population with and without pemphigus and the population with and without pemphigoid were matched according to age, gender and race using propensity score matching (PSM) with a 1:2 ratio of cases: controls. Odds ratios (ORs) and 95% confidence intervals (CIs) for the associations between the study variables and AD were analyzed using logistic regression analysis with the PROC SURVEYLOGISTIC procedure. Covariates that remained significantly different between case and control groups after matching were identified and were adjusted for multivariable regression analyses. All *p*-values were two-sided and *p* < 0.05 was considered statistical significance. All statistical analyses were performed using the statistical software package SAS software version 9.4 (SAS Institute Inc., Cary, NC, USA).

## Results

### Study population

**Fig. 1:**
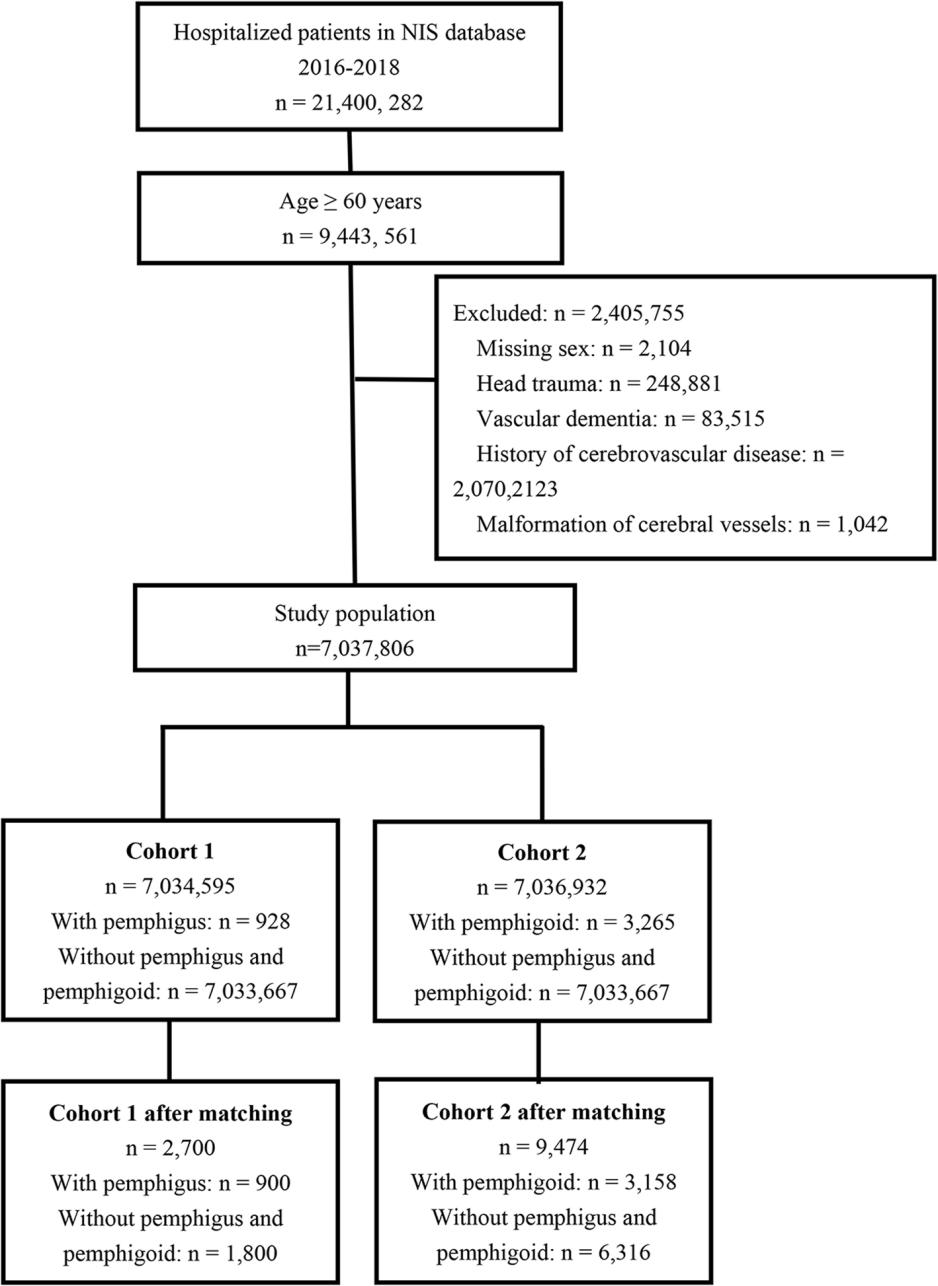
Flow diagram of study population selection

The flow diagram of study population selection is shown in Fig. 1. The present study extracted data of 9,443,561 hospitalized adults aged ≥60 years from the NIS database during 2016 to 2018. Subjects with missing information of sex, diagnosed with head trauma, vascular dementia, malformation of cerebral vessels, or had a history of cerebrovascular diseases were excluded (*n* = 2,405,755). A total of 7,037,806 older adult subjects were included as the study population, of whom 7,034,595 formed Cohort 1, including928 with pemphigus and 7,033,667 without pemphigus or pemphigoid. After PSM, 2700 subjects remained, including 900 with pemphigus and 1800 without pemphigus or pemphigoid. A total of 7,036,932 subjects formed Cohort 2, which included 3265 with pemphigoid and 7,033,667 without pemphigus or pemphigoid. After PSM, 9474 individuals remained, including 3158 with pemphigoid and 6316 without pemphigus or pemphigoid.

### Characteristics of subjects with and without pemphigus

Characteristics of subjects with and without pemphigus are summarized in Table 1. Subjects’ mean age was 73.9 ± 0.02 years, and more than half were females (53.2%). The majority of the cohort were White (76.3%), with insurance covered by Medicare or Medicaid (82.2%), and without active tobacco use (63.0%) or excessive alcohol use (96.4%).

**Table 1:** Characteristics of patients with and without pemphigus

Characteristics	Before matching				After matching			
	Total (*n* = 7,034,595)	With pemphigus (*n* = 928)	Without pemphigus (*n* = 7,033,667) ^a^	*p*-value	Total (*n* = 2700)	With pemphigus (*n* = 900)	Without pemphigus (*n* = 1800) ^a^	*p*-value
**AD**	175,291 (2.5)	43 (4.6)	175,248 (2.5)	**< 0.001**	90 (3.3)	41 (4.6)	49 (2.7)	**0.011**
**Age**	73.9 ± 0.02	76.9 ± 0.30	73.9 ± 0.02	**< 0.001**	76.8 ± 0.2	77.0 ± 0.3	76.7 ± 0.2	0.425
60–69	2,660,011 (37.8)	236 (25.4)	2,659,775 (37.8)	**< 0.001**	675 (25.0)	225 (25.0)	450 (25.0)	1.000
70–79	2,310,666 (32.9)	298 (32.1)	2,310,368 (32.9)		864 (32.0)	288 (32.0)	576 (32.0)	
80+	2,063,918 (29.3)	394 (42.5)	2,063,524 (29.3)		1161 (43.0)	387 (43.0)	774 (43.0)	
Sex				0.325				1.000
Male	3,295,232 (46.8)	419 (45.2)	3,294,813 (46.8)		1215 (45.0)	405 (45.0)	810 (45.0)	
Female	3,739,363 (53.2)	509 (54.9)	3,738,854 (53.2)		1485 (55.0)	495 (55.0)	990 (55.0)	
**Race**				**< 0.001**				1.000
White	5,199,571 (76.3)	651 (72.3)	5,198,920 (76.3)		1953 (72.3)	651 (72.3)	1302 (72.3)	
Black	728,534 (10.7)	85 (9.4)	728,449 (10.7)		255 (9.4)	85 (9.4)	170 (9.4)	
Hispanic	530,188 (7.8)	86 (9.6)	530,102 (7.8)		258 (9.6)	86 (9.6)	172 (9.6)	
Others	356,397 (5.2)	78 (8.7)	356,319 (5.2)		234 (8.7)	78 (8.7)	156 (8.7)	
Missing	219,905	28	219,877		0	0	0	
**Household income**				**< 0.001**				**0.018**
Quartile1	1,942,389 (28.1)	213 (23.2)	1,942,176 (28.1)		679 (25.6)	206 (23.2)	473 (26.8)	
Quartile2	1,856,586 (26.8)	253 (27.6)	1,856,333 (26.8)		708 (26.7)	244 (27.4)	464 (26.3)	
Quartile3	1,684,484 (24.4)	214 (23.3)	1,684,270 (24.4)		655 (24.7)	206 (23.2)	449 (25.4)	
Quartile4	1,434,891 (20.7)	237 (25.9)	1,434,654 (20.7)		612 (23.1)	233 (26.2)	379 (21.5)	
Missing	116,245	11	116,234		46	11	35	
**Insurance status**				**< 0.001**				0.120
Medicare/Medicaid	5,778,056 (82.2)	821 (88.6)	5,777,235 (82.2)		2343 (86.8)	797 (88.7)	1546 (85.9)	
Private including HMO	1,016,340 (14.5)	89 (9.6)	1,016,251 (14.5)		293 (10.9)	85 (9.5)	208 (11.6)	
Self-pay/no-charge/other	232,118 (3.3)	17 (1.8)	232,101 (3.3)		62 (2.3)	17 (1.9)	45 (2.5)	
Missing	8081	1	8080		2	1	1	
**Active tobacco use**				**< 0.001**				**< 0.001**
No	4,428,686 (63.0)	675 (72.7)	4,428,011 (63.0)		1803 (66.8)	653 (72.6)	1150 (63.9)	
Yes	2,605,909 (37.0)	253 (27.3)	2,605,656 (37.1)		897 (33.2)	247 (27.4)	650 (36.1)	
**Excessive alcohol use**				**< 0.001**				**0.012**
No	6,784,456 (96.4)	916 (98.7)	6,783,540 (96.4)		2637 (97.7)	888 (98.7)	1749 (97.2)	
Yes	250,139 (3.6)	12 (1.3)	250,127 (3.6)		63 (2.3)	12 (1.3)	51 (2.8)	
**Chronic disease**								
Ischemic heart disease	2,173,048 (30.9)	241 (26.0)	2,172,807 (30.9)	**0.002**	795 (29.4)	234 (26.0)	561 (31.2)	**0.006**
Valvular heart disease	245,990 (3.5)	35 (3.8)	245,955 (3.5)	0.662	97 (3.6)	35 (3.9)	62 (3.4)	0.565
DM	2,408,768 (34.2)	367 (39.6)	2,408,401 (34.2)	**0.001**	945 (35.0)	356 (39.6)	589 (32.7)	**< 0.001**
Hypertension	5,282,711 (75.1)	684 (73.7)	5,282,027 (75.1)	0.326	2018 (74.7)	664 (73.8)	1354 (75.2)	0.402
Dyslipidemia	3,231,935 (45.9)	391 (42.1)	3,231,544 (45.9)	**0.026**	1191 (44.1)	379 (42.1)	812 (45.1)	0.145
Obesity	1,200,899 (17.1)	170 (18.3)	1,200,729 (17.1)	0.316	425 (15.7)	163 (18.1)	262 (14.6)	**0.013**
Chronic pulmonary disease	2,051,208 (29.2)	207 (22.3)	2,051,001 (29.2)	**< 0.001**	735 (27.2)	200 (22.2)	535 (29.7)	**< 0.001**
Severe liver disease	112,867 (1.6)	3 (0.3)	112,864 (1.6)	**0.002**	29 (1.1)	3 (0.3)	26 (1.4)	**< 0.001**
Moderate to severe kidney disease	299,375 (4.3)	28 (3.0)	299,347 (4.3)	0.071	104 (3.9)	27 (3.0)	77 (4.3)	0.113
Coagulopathy	517,828 (7.4)	89 (9.6)	517,739 (7.4)	**0.010**	229 (8.5)	87 (9.7)	142 (7.9)	0.116
Peripheral vascular disease	726,322 (10.3)	77 (8.3)	726,245 (10.3)	**0.048**	271 (10.0)	76 (8.4)	195 (10.8)	0.048
Atrial fibrillation	1,698,443 (24.1)	248 (26.7)	1,698,195 (24.1)	0.084	760 (28.1)	240 (26.7)	520 (28.9)	0.237
Psychosis	105,568 (1.5)	13 (1.4)	105,555 (1.5)	0.836	36 (1.3)	13 (1.4)	23 (1.3)	0.755
Depression	932,319 (13.3)	130 (14.0)	932,189 (13.3)	0.504	360 (13.3)	127 (14.1)	233 (12.9)	0.395
**Systemic autoimmune disease**								
SLE	27,637 (0.4)	7 (0.8)	27,630 (0.4)	0.078	12 (0.4)	7 (0.8)	5 (0.3)	0.058
RA	186,468 (2.7)	36 (3.9)	186,432 (2.7)	**0.027**	79 (2.9)	34 (3.8)	45 (2.5)	0.058
IBD	61,684 (0.9)	6 (0.7)	61,678 (0.9)	0.515	16 (0.6)	5 (0.6)	11 (0.6)	0.874
SSc	9270 (0.1)		9270 (0.1)		3 (0.1)	0 (0.0)	3 (0.2)	
Sjogren’s syndrome	15,759 (0.2)	1 (0.1)	15,758 (0.2)	0.453	6 (0.2)	1 (0.1)	5 (0.3)	0.377
**Other chronic inflammatory skin disease**								
Atopic dermatitis	1359 (0.02)	1 (0.11)	1358 (0.02)	0.053	1 (0.0)	1 (0.1)	0 (0.0)	
Hidradenitis	1571 (0.02)	0 (0.00)	1571 (0.02)		2 (0.1)	0 (0.0)	2 (0.1)	
Dermatomyositis & polymyositis	3651 (0.1)	2 (0.2)	3649 (0.1)	**0.029**	2 (0.1)	2 (0.2)	0 (0.0)	
Psoriasis	43,822 (0.6)	12 (1.3)	43,810 (0.6)	**0.009**	19 (0.7)	11 (1.2)	8 (0.4)	**0.010**
Alopecia areata	133 (0.0)	0 (0.0)	133 (0.0)		0 (0.0)	0 (0.0)	0 (0.0)	
Vitiligo	1815 (0.03)	0 (0.0)	1815 (0.03)		0 (0.0)	0 (0.0)	0 (0.0)	
Pyoderma gangrenosum	1272 (0.02)	2 (0.22)	1270 (0.02)	**< 0.001**	2 (0.1)	2 (0.2)	0 (0.0)	
Rosacea	6639 (0.1)	1 (0.1)	6638 (0.1)	0.894	2 (0.1)	1 (0.1)	1 (0.1)	0.479
**Hospital bed size**				0.278				0.081
Large	3,415,536 (48.6)	476 (51.3)	3,415,060 (48.6)		1302 (48.2)	461 (51.2)	841 (46.7)	
Medium	2,083,258 (29.6)	259 (27.9)	2,082,999 (29.6)		817 (30.3)	253 (28.1)	564 (31.3)	
Small	1,535,801 (21.8)	193 (20.8)	1,535,608 (21.8)		581 (21.5)	186 (20.7)	395 (21.9)	
**Hospital location/teaching status**				**0.049**				**0.016**
Urban teaching	4,514,785 (64.2)	632 (68.1)	4,514,153 (64.2)		1760 (65.2)	616 (68.4)	1144 (63.6)	
Urban nonteaching	1,768,762 (25.1)	213 (23.0)	1,768,549 (25.1)		663 (24.6)	209 (23.2)	454 (25.2)	
Rural	751,048 (10.7)	83 (8.9)	750,965 (10.7)		277 (10.3)	75 (8.3)	202 (11.2)	
**Hospital region**				**< 0.001**				**0.019**
Northeast	1,383,332 (19.7)	238 (25.7)	1,383,094 (19.7)		629 (23.3)	237 (26.3)	392 (21.8)	
Midwest	1,581,671 (22.5)	184 (19.8)	1,581,487 (22.5)		517 (19.1)	165 (18.3)	352 (19.6)	
South	2,757,811 (39.2)	305 (32.9)	2,757,506 (39.2)		993 (36.8)	302 (33.6)	691 (38.4)	
West	1,311,781 (18.7)	201 (21.7)	1,311,580 (18.7)		561 (20.8)	196 (21.8)	365 (20.3)	

Continuous variables are presented as mean ± SE; categorical variables are presented as unweighted counts (weighted percentage). *P*-value < 0.05 are showed in bold

*Abbreviations*: *AD* Alzheimer’s disease, *DM* diabetes mellitus, *SLE* systemic lupus erythematosus, *RA* rheumatoid arthritis, *IBD* inflammatory bowel disease, *SSc* systemic sclerosis

^a^ Excluded pemphigoid

After matching, subjects with pemphigus had a higher percentage of fourth quartile household income (Quartile 4: 26.2% vs. 21.5%, *p* = 0.018), lower (no) active tobacco use (72.6% vs. 63.9%, *p* < 0.001) and lower (no) excessive alcohol use (98.7% vs. 97.2%, *p* = 0.012) than those without pemphigus. Regarding comorbid chronic conditions, subjects with pemphigus had higher percentages of DM (39.6% vs. 32.7%, *p* < 0.001), obesity (18.1% vs. 14.6%, *p* = 0.013) and psoriasis (1.2% vs. 0.4%, *p* = 0.010). A significantly greater percentage of older adults with pemphigus had AD (4.6% vs. 2.7%, *p* = 0.011) than among those who had no pemphigus.

### Characteristics of subjects with and without pemphigoid

Characteristics of older adult subjects with or without pemphigoid are summarized in Table 2. After matching, subjects with pemphigoid had a higher percentage of third quartile household income (Quartile 3: 25.5% vs. 24.6%, Quartile 4: 29.5% vs. 22.5%, *p* < 0.001), insurance covered by Medicare or Medicaid (91.6% vs. 88.9%, *p* < 0.001), and excessive alcohol use (2.6% vs. 2.2%, *p* < 0.001) than those without pemphigoid. Regarding comorbid chronic conditions, subjects with pemphigoid had higher percentages of DM (43.9% vs. 32.6%, *p* < 0.001), hypertension (80.8% vs. 77.2%, *p* < 0.001), obesity (21.9% vs. 14.0%, *p* < 0.001), coagulopathy (9.3% vs. 7.9%, *p* = 0.017) and psoriasis (1.9% vs. 0.6%, *p* < 0.001) than those without pemphigoid. Subjects with pemphigoid had a significantly higher percentage of AD (4.7% vs. 3.4%, *p* = 0.003) than those without pemphigoid.

**Table 2:** Characteristics of patients with and without pemphigoid

Characteristics	Before matching				After matching			
	Total (*n* = 7,036,932)	With pemphigoid (*n* = 3265)	Without pemphigoid (*n* = 7,033,667) ^a^	*p*-value	Total (*n* = 9474)	With pemphigoid (*n* = 3158)	Without pemphigoid (*n* = 6316) ^a^	*p*-value
**AD**	175,398 (2.5)	150 (4.6)	175,248 (2.5)	**< 0.001**	363 (3.8)	147 (4.7)	216 (3.4)	**0.003**
**Age, years**	73.9 ± 0.02	79.2 ± 0.16	73.9 ± 0.02	**< 0.001**	78.8 ± 0.1	79.2 ± 0.2	78.6 ± 0.1	**0.003**
60–69	2,660,325 (37.8)	550 (16.9)	2,659,775 (37.8)	**< 0.001**	1584 (16.7)	528 (16.7)	1056 (16.7)	1.000
70–79	2,311,346 (32.9)	978 (30.0)	2,310,368 (32.9)		2841 (30.0)	947 (30.0)	1894 (30.0)	
80+	2,065,261 (29.4)	1737 (53.2)	2,063,524 (29.3)		5049 (53.3)	1683 (53.3)	3366 (53.3)	
**Sex**				0.680				1.000
Male	3,296,330 (46.8)	1517 (46.5)	3,294,813 (46.8)		4392 (46.4)	1464 (46.4)	2928 (46.4)	
Female	3,740,602 (53.2)	1748 (53.5)	3,738,854 (53.2)		5082 (53.6)	1694 (53.6)	3388 (53.6)	
**Race**				**< 0.001**				1.000
White	5,201,334 (76.3)	2414 (76.4)	5,198,920 (76.3)		7242 (76.4)	2414 (76.4)	4828 (76.4)	
Black	728,794 (10.7)	345 (10.9)	728,449 (10.7)		1035 (10.9)	345 (10.9)	690 (10.9)	
Hispanic	530,265 (7.8)	163 (5.2)	530,102 (7.8)		489 (5.2)	163 (5.2)	326 (5.2)	
Others	356,555 (5.2)	236 (7.5)	356,319 (5.2)		708 (7.5)	236 (7.5)	472 (7.5)	
Missing	219,984	107	219,877		0	0	0	
**Household income**				**< 0.001**				**< 0.001**
Quartile1	1,942,868 (28.1)	692 (21.4)	1,942,176 (28.1)		2311 (24.7)	678 (21.7)	1633 (26.3)	
Quartile2	1,857,101 (26.8)	768 (23.8)	1,856,333 (26.8)		2386 (25.5)	727 (23.3)	1659 (26.7)	
Quartile3	1,685,094 (24.4)	824 (25.5)	1,684,270 (24.4)		2324 (24.9)	796 (25.5)	1528 (24.6)	
Quartile4	1,435,599 (20.7)	945 (29.3)	1,434,654 (20.7)		2318 (24.8)	921 (29.5)	1397 (22.5)	
Missing	116,270	36	116,234		135	36	99	
**Insurance status**				**< 0.001**				**< 0.001**
Medicare/Medicaid	5,780,220 (82.2)	2985 (91.5)	5,777,235 (82.2)		8498 (89.8)	2890 (91.6)	5608 (88.9)	
Private including HMO	1,016,463 (14.5)	212 (6.5)	1,016,251 (14.5)		780 (8.2)	206 (6.5)	574 (9.1)	
Self-pay/no-charge/other	232,166 (3.3)	65 (2.0)	232,101 (3.3)		189 (2.0)	60 (1.9)	129 (2.0)	
Missing	8083	3	8080		7	2	5	
**Active tobacco use**				**< 0.001**				0.189
No	4,430,321 (63.0)	2310 (70.8)	4,428,011 (63.0)		6355 (67.1)	2231 (70.6)	4124 (65.3)	
Yes	2,606,611 (37.0)	955 (29.3)	2,605,656 (37.1)		3119 (32.9)	927 (29.4)	2192 (34.7)	
**Excessive alcohol use**				**0.007**				**< 0.001**
No	6,786,718 (96.4)	3178 (97.3)	6,783,540 (96.4)		9255 (97.7)	3076 (97.4)	6179 (97.8)	
Yes	250,214 (3.6)	87 (2.7)	250,127 (3.6)		219 (2.3)	82 (2.6)	137 (2.2)	
**Chronic disease**								
Ischemic heart disease	2,173,764 (30.9)	957 (29.3)	2,172,807 (30.9)	0.056	3049 (32.2)	936 (29.6)	2113 (33.5)	**< 0.001**
Valvular heart disease	246,112 (3.5)	157 (4.8)	245,955 (3.5)	**< 0.001**	424 (4.5)	151 (4.8)	273 (4.3)	0.336
DM	2,409,835 (34.3)	1434 (43.9)	2,408,401 (34.2)	**< 0.001**	3446 (36.4)	1385 (43.9)	2061 (32.6)	**< 0.001**
Hypertension	5,284,662 (75.1)	2635 (80.7)	5,282,027 (75.1)	**< 0.001**	7429 (78.4)	2551 (80.8)	4878 (77.2)	**< 0.001**
Dyslipidemia	3,233,028 (45.9)	1484 (45.5)	3,231,544 (45.9)	0.586	4400 (46.4)	1438 (45.5)	2962 (46.9)	0.214
Obesity	1,201,438 (17.1)	709 (21.7)	1,200,729 (17.1)	**< 0.001**	1577 (16.6)	691 (21.9)	886 (14.0)	**< 0.001**
Chronic pulmonary disease	2,051,952 (29.2)	951 (29.1)	2,051,001 (29.2)	0.968	2744 (29.0)	914 (28.9)	1830 (29.0)	0.975
Severe liver disease	112,892 (1.6)	28 (0.9)	112,864 (1.6)	**0.001**	96 (1.0)	27 (0.9)	69 (1.1)	0.297
Moderate to severe kidney disease	299,502 (4.3)	155 (4.8)	299,347 (4.3)	0.211	390 (4.1)	149 (4.7)	241 (3.8)	0.053
Coagulopathy	518,045 (7.4)	306 (9.4)	517,739 (7.4)	**< 0.001**	790 (8.3)	294 (9.3)	496 (7.9)	**0.017**
Peripheral vascular disease	726,601 (10.3)	356 (10.9)	726,245 (10.3)	0.289	1086 (11.5)	345 (10.9)	741 (11.7)	0.249
Atrial fibrillation	1,699,223 (24.2)	1028 (31.4)	1,698,195 (24.1)	**< 0.001**	2935 (31.0)	993 (31.4)	1942 (30.7)	0.501
Psychosis	105,599 (1.5)	44 (1.4)	105,555 (1.5)	0.481	110 (1.2)	43 (1.4)	67 (1.1)	0.201
Depression	932,607 (13.3)	418 (12.8)	932,189 (13.3)	0.462	1121 (11.8)	403 (12.8)	718 (11.4)	0.053
**Systemic autoimmune disease**								
SLE	27,644 (0.4)	14 (0.4)	27,630 (0.4)	0.758	32 (0.3)	14 (0.4)	18 (0.3)	0.215
RA	186,516 (2.7)	84 (2.6)	186,432 (2.7)	0.793	247 (2.6)	79 (2.5)	168 (2.7)	0.653
IBD	61,701 (0.9)	23 (0.7)	61,678 (0.9)	0.311	76 (0.8)	23 (0.7)	53 (0.8)	0.581
SSc	9273 (0.1)	3 (0.1)	9270 (0.1)	0.529	12 (0.1)	3 (0.1)	9 (0.1)	0.539
Sjogren’s syndrome	15,770 (0.2)	12 (0.4)	15,758 (0.2)	0.108	24 (0.3)	12 (0.4)	12 (0.2)	0.090
**Other chronic inflammatory skin disease**								
Atopic dermatitis	1361 (0.02)	3 (0.09)	1358 (0.02)	**0.003**	3 (0.0)	3 (0.1)	0 (0.0)	
Hidradenitis	1572 (0.02)	1 (0.03)	1571 (0.02)	0.751	4 (0.0)	1 (0.0)	3 (0.0)	0.722
Dermatomyositis & polymyositis	3652 (0.1)	3 (0.1)	3649 (0.1)	0.316	7 (0.1)	3 (0.1)	4 (0.1)	0.592
Psoriasis	43,873 (0.6)	63 (1.9)	43,810 (0.6)	**< 0.001**	96 (1.0)	59 (1.9)	37 (0.6)	**< 0.001**
Alopecia areata	134 (0.00)	1 (0.03)	133 (0.00)	**< 0.001**	1 (0.0)	1 (0.0)	0 (0.0)	
Vitiligo	1817 (0.03)	2 (0.06)	1815 (0.03)	0.208	2 (0.0)	2 (0.1)	0 (0.0)	
Pyoderma gangrenosum	1274 (0.02)	4 (0.12)	1270 (0.02)	**< 0.001**	4 (0.0)	4 (0.1)	0 (0.0)	
Rosacea	6642 (0.1)	4 (0.1)	6638 (0.1)	0.600	7 (0.1)	4 (0.1)	3 (0.0)	0.181
**Hospital bed size**				0.183				0.119
Large	3,416,691 (48.6)	1631 (50.0)	3,415,060 (48.6)		4568 (48.2)	1567 (49.6)	3001 (47.5)	
Medium	2,083,915 (29.6)	916 (28.1)	2,082,999 (29.6)		2792 (29.5)	890 (28.2)	1902 (30.1)	
Small	1,536,326 (21.8)	718 (22.0)	1,535,608 (21.8)		2114 (22.3)	701 (22.2)	1413 (22.4)	
**Hospital location/teaching status**				**< 0.001**				**< 0.001**
Urban teaching	4,516,510 (64.2)	2357 (72.2)	4,514,153 (64.2)		6248 (65.9)	2275 (72.0)	3973 (62.9)	
Urban nonteaching	1,769,201 (25.1)	652 (20.0)	1,768,549 (25.1)		2265 (23.9)	640 (20.3)	1625 (25.7)	
Rural	751,221 (10.7)	256 (7.8)	750,965 (10.7)		961 (10.1)	243 (7.7)	718 (11.4)	
**Hospital region**				**< 0.001**				**< 0.001**
Northeast	1,383,999 (19.7)	905 (27.7)	1,383,094 (19.7)		2272 (24.0)	891 (28.2)	1381 (21.9)	
Midwest	1,582,288 (22.5)	801 (24.5)	1,581,487 (22.5)		2114 (22.3)	747 (23.7)	1367 (21.6)	
South	2,758,422 (39.2)	916 (28.1)	2,757,506 (39.2)		3306 (34.9)	900 (28.5)	2406 (38.1)	
West	1,312,223 (18.7)	643 (19.7)	1,311,580 (18.7)		1782 (18.8)	620 (19.6)	1162 (18.4)	

All significant values are shown in bold

*Abbreviation*: *AD* Alzheimer’s disease, *DM* diabetes mellitus, *SLE* systemic lupus erythematosus, *RA* rheumatoid arthritis, *IBD* inflammatory bowel disease, *SSc* systemic sclerosis

^a^Excluded pemphigus

### Associations between pemphigus and AD

Table 3 summarizes the results of univariate and multivariable analyses of associations between pemphigus and AD. After adjusting for age, active tobacco use, obesity and chronic pulmonary disease, the odds of AD were significantly higher in subjects with pemphigus (adjusted OR [aOR], 1.69, 95% CI: 1.10–2.57) than those for subjects without pemphigus. In further subgroup analyses, significant associations were observed between pemphigus and AD among subjects aged over 80 years (aOR, 1.72, 95% CI: 1.08–2.75) but not among those aged 60–79 or 70–79 years. Moreover, female subjects with pemphigus were significantly more likely to have AD (aOR, 1.78, 95% CI: 1.02–3.12), but not males.

**Table 3:** Associations between pemphigus and AD

	AD	
	Crude OR (95% CI)	Adjusted OR (95% CI)
Overall ^a^	**1.71 (1.12, 2.59)**	**1.69 (1.10, 2.57)**
Subgroups		
Age, years ^b^		
60–69	2.00 (0.12, 32.42)	2.78 (0.10, 81.44)
70–79	1.57 (0.63, 3.89)	1.48 (0.59, 3.73)
80+	**1.76 (1.10, 2.81)**	**1.72 (1.08, 2.75)**
Sex ^c^		
Male	1.63 (0.93, 2.86)	1.58 (0.89, 2.80)
Female	**1.78 (1.02, 3.11)**	**1.78 (1.02, 3.12)**
Race ^d^		
White	1.56 (0.96, 2.54)	1.55 (0.95, 2.53)
Black	1.00 (0.19, 5.31)	0.97 (0.19, 5.03)
Hispanic	2.75 (0.90, 8.41)	2.99 (0.93, 9.60)

Significant values are shown in bold

*AD* Alzheimer’s disease, *OR* odds ratio, *CI* confidence interval

^a c, d^ Adjusted for age, active tobacco use, obesity and chronic pulmonary disease

^b^ Adjusted for active tobacco use, obesity and chronic pulmonary disease

### Associations between pemphigoid and AD

Table 4 displays the results of univariate and multivariable analyses of associations between pemphigoid and AD. After adjusting for age, insurance status, active tobacco use, obesity, chronic pulmonary disease, moderate-to- severe kidney disease and depression, pemphigoid was independently associated with the presence of AD (aOR, 1.39, 95% CI: 1.12–1.73). In subgroup analyses, when stratified by age, significant associations were observed between pemphigoid and AD among subjects aged over 80 years (aOR, 1.40, 95% CI: 1.10–1.78) but not among those aged 60–79 or 70–79 years. In addition, when stratified by sex, associations between pemphigoid and AD were significant in females (aOR, 1.63, 95% CI: 1.23–2.15) but not in males. The associations between pemphigoid and AD were significantly higher in Hispanics (aOR, 3.13, 95% CI: 1.14–8.59) than in Whites (aOR, 1.21, CI: 0.94–1.56) or Blacks (aOR, 1.53 95% CI: 0.84–2.78).

**Table 4:** Associations between pemphigoid and AD

Characteristic	AD	
	Crude OR (95% CI)	Adjusted OR (95% CI)
Overall ^a^	**1.38 (1.11, 1.71)**	**1.39 (1.12, 1.73)**
Subgroups		
Age, years^b^		
60–69	3.01 (0.50, 18.09)	2.18 (0.36, 13.09)
70–79	1.22 (0.73, 2.03)	1.27 (0.76, 2.13)
80+	**1.40 (1.10, 1.79)**	**1.40 (1.10, 1.78)**
Sex ^c^		
Male	1.14 (0.82, 1.58)	1.11 (0.80, 1.55)
Female	**1.59 (1.20, 2.10)**	**1.63 (1.23, 2.15)**
Race ^d^		
White	1.20 (0.93, 1.55)	1.21 (0.94, 1.56)
Black	1.53 (0.85, 2.73)	1.53 (0.84, 2.78)
Hispanic	**3.45 (1.33, 8.95)**	**3.13 (1.14, 8.59)**

Significant values are shown in bold

*AD* Alzheimer’s disease, *OR* odds ratio, *CI* confidence interval

^a c, d^ Adjusted for age group, insurance status, active tobacco use, obesity, chronic pulmonary disease, moderate to severe kidney disease and depression

^b^ Adjusted for insurance status, active tobacco use obesity, chronic pulmonary disease, moderate to severe kidney disease and depression

## Discussion

The present population-based cross-sectional study is the first to assess associations between the skin disorders pemphigus and pemphigoid and AD among adults aged 60 years and older. Results showed that pemphigus was independently associated with an increased risk (69%) of developing AD. In particular, adults aged 80 years and older with pemphigus were 72% more likely to develop AD than adults without pemphigus. In addition, women with pemphigus were 78% more likely to develop AD than women without pemphigus. Similarly, but to a lesser extent, pemphigoid was independently associated with a 39% increased risk for AD and individuals with pemphigoid aged 80 years or older were 40% more likely to have AD than those without pemphigoid.

Several previous studies have noted a relationship between AD and skin disorders. In patients with AD, the physiology of the skin is altered, and proteins associated with neurodegenerative disease have been detected [26]. The risk of developing pemphigoid is also reported to be significantly increased in patients with AD [24]. Pemphigus and pemphigoid are systemic immune-mediated disorders rather than isolated cutaneous immune disorders, and may be associated with neurologic disorders. Together pemphigus and bullous pemphigoid represent a significant disease burden and unmet medical need, with higher costs due to increased hospitalizations and a lack of adequate treatment [27]. The possible association with neurological disease adds to the health and economic burden, empasizing a need for further investigation.

Bullous pemphigoid is the most common subcutaneous autoimmune blistering disease, with increased incidence in older adults. Several previous studies have reported associations between pemphigoid and neurological disorders [28–31]. A previous single-center cohort study assessed associations between blood pressure and neurologic disease in patients with pemphigoid, finding significant associations between pemphigoid and neurologic diseases, including dementia, Parkinson’s disease, and stroke, while AD was notably not significantly associated [31]. Another previous study included one of the largest cohorts of pemphigus patients to estimate associations between pemphigus and four neurological disorders [29]. Those authors observed associations between pemphigus and dementia, Parkinson’s disease, and epilepsy, and suggested that treating physicians should carefully evaluate patients with pemphigus for coexisting neurologic disorders in order to provide appropriate treatment. A previous systematic review described the occurrence of neurologic disease specifically in patients with pemphigoid, noting increased risk of stroke, dementia, Parkinson’s disease, and epilepsy [27]. Neurologic disease preceded pemphigoid in most cases and the increased associations with neurologic disease were associated with increased mortality.

The bullous pemphigoid antibody targets two epidermal adhesion molecules, BP180 and BP230. Homologues of these proteins have now been identified in the brain, and neurological disorders have been hypothesized to lead to the production of autoantibodies that can cross-react with their skin forms [32, 33]. Bullous pemphigoid is characterized by self-production of IgG against two self-antigens including BP180 and BP230, which are epidermal adhesion molecules not only expressed in the skin, but also widely present in brain neurons. The presence of proteins shared between the central nervous system and the skin are recognized by self-antigens in the central nervous system, and neuroinflammation may disrupt the blood-brain barrier, leading to cross-reactive immune reactions between skin and brain self-antigens and the ability to cause neurological disease [34]. Nevertheless, data to refute this theory are mounting. A recent study was unable to locate BP180 in the hippocampus of the human brain [35]. Other authors found little evidence that BP180 is expressed in the brains of healthy mice or humans [36].

Preliminary observations in the clinic indicate that subjective neurocognitive complaints are relatively common in patients with bullous pemphigoid. A previous multicenter observational case-control study noted cognitive decline and a higher risk of developing cognitive impairment in patients with pemphigoid [20]. Those authors suggested that assessment of cognitive impairment in such patients may be required in clinical practice for early diagnosis and treatment of dementia. Another previous retrospective nationwide study using the Finnish Healthcare Diagnostic Register from 1987 to 2013 showed that patients with pemphigoid had an increased risk of vascular dementia and AD [18]. It must be noted that AD and vascular dementia are two different types of dementia. Although some investigators have suggested that pemphigus and pemphigoid are associated with dementia risk, AD has rarely been studied as an isolated subtype of dementia. The present study excluded vascular dementia and related vascular conditions in order to better observe the associations between pemphigus/pemphigoid and AD.

The mechanisms underlying the associations found between pemphigus, pemphigoid, and AD in our analysis are likely multifaceted. This connection might be due to inflammation, given that both pemphigus and pemphigoid are inflammatory autoimmune skin conditions. The systemic inflammation linked to these conditions might potentially contribute to the development of AD [37]. Further, genetic factors, immune responses, chronic stress, and shared risk factors might also be at play. Our analysis revealed notably increased odds of AD in association with pemphigus/pemphigoid among individuals aged 80 years or older, rather than among those aged 60–79 years. This finding is intersting, especially considering that pemphigus is most prevalent in adults 40–50 years of age. This could be elucidated by considering several hypotheses. Elderly individuals aged 80 years or above might experience a longer duration of pemphigus or pemphigoid, exposing their bodies to systemic inflammation and chronic stress for a longer period. This prolonged exposure could potentially accumulate the risk of developing AD. Nevertheless, it’s crucial to note that our present study cannot establish a direct causal relationship due to the nature of study design. Therefore, further research is highly recommended to delve deeper into these associations and to address the underlying mechanisms.

## Limitations

The present study has several limitations. First, this is not a longitudinal analysis, and the diagnosis date or duration of AD and pemphigus/pemphigoid was not available. This limitation restricts our ability to establish causal relationships. The study lacks data concerning the subtypes of pemphigus (vulgaris, foliaceus, or paraneoplastic), and severity of both pemphigus and pemphigoid in the study population. Factors such as education, lifestyle, physical activity, and prescribed medications, may also contribute to the observed association; however, these factors were not included in the NIS database. Individuals’ genetic profiles may be important, but also were not captured. Nevertheless, while the possibility of miscoding exists as any research based on claim data, it’s important to note that the codes utilized in this study have undergone prior validation. Therefore, any potential bias stemming from miscoding is likely to be minimal due to the reliability of the established codes.

## Conclusions

Results of this population-based, cross-sectional study show that pemphigus and pemphigoid are independently associated with AD in older adults in the US, especially among females and octogenarians. Further studies addressing the etiology and the mechanisms underlying pemphigus and pemphigoid and their associations with AD are highly warranted.

## Data Availability

The datasets analysed during the current study are available from the corresponding author on reasonable request.

## Abbreviations

AD: Alzheimer’s disease
NIS: Nationwide Inpatient Sample
Aβ: amyloid-β peptide
Dsg: desmoglein
HCUP: Healthcare Cost and Utilization Project
NIH: National Institutes of Health
PSM: propensity score matching
ORs: Odds ratios
CIs: confidence intervals
